# Bullying victimization and its associated factors among adolescents in Kozhikode district, Kerala, India: a mixed-methods study [version 1; peer review: 1 approved]

**DOI:** 10.12688/wellcomeopenres.17102.1

**Published:** 2021-09-07

**Authors:** Bhagiaswari Kodapally, Elezebeth Mathews, Prakash Babu Kodali, Kavumpurathu Raman Thankappan

**Affiliations:** Department of Public Health and Community Medicine, Central University of Kerala, Kasargod, Kerala, 671315, India

**Keywords:** Bullying, Cyberbullying, Schools, School Health Services, Victimisation

## Abstract

**Background:**

Bullying victimization among adolescents is a serious concern as it leads to poor psycho-social adjustments in the future. Literature on bullying at schools in Kerala is limited. This study aimed to investigate the magnitude of bullying and the factors associated with it among adolescents.

**Methods:**

A sequential explanatory study design was used. A cross-sectional study among 764 adolescents (mean age 13.3 years, males 58.5%) selected through multistage cluster sampling was done. We used the Olweus Bully-Victim Questionnaire, Global School Health Survey, and Patient Health Questionnaire 9 for data collection. Binary logistic regression was performed to identify predictors of bullying victimization. After this, in-depth interviews were carried out among key stakeholders.

**Results:**

About 117 (15.3%) respondents reported being bullied at least twice a month. Verbal bullying was reported by 30% (n=229), physical bullying by 23.3% (n=178), sexual bullying by 11% (n=89) and cyber-bullying by 3.3% (n=25). Adolescents aged 14 years and above (OR 2.09, 95% CI: 1.34-3.26), being male (OR 3.50, 95% CI: 1.97-5.87), the parent’s response to bullying (OR 5.27, 95% CI: 2.44-11.36), the victim’s reaction to being bullied (OR 8.101, CI: 4.53-14.36) and the teacher’s action against the bully (OR 3.59, CI: 1.91-6.73) were major predictors of bullying. Qualitative exploration of pathways of bullying phenomena revealed the influence of parenting on a child being a victim or a bully, and a lack of competence and training among teachers to address bullying at school.

**Conclusions:**

Bullying is highly prevalent among adolescents in schools and has short- and long-term implications. Targeted interventions for bullying prevention should focus on older adolescent boys and those who report being bullied. Anti-bullying policies at school are vital to sensitize teachers, parents, and students to bullying.

## Introduction

Bullying is a significant problem which adolescents and children face, with a prevalence ranging from 7% to 74% globally (UNESCO, 2018). The consequences of bullying range from short-term effects like anxiety, depression, and self-harm to long-term impacts such as post-traumatic stress disorder (PTSD), personality disorders, and suicidal tendencies. Bullying refers to aggressive behaviour that is repetitive and intentional, where a power differential exists between two people (Rettew & Pawlowski, 2016). The practice of bullying often involves three phenomena: i) the bully or the perpetrator, ii) the ones bullied or bully-victims, and iii) and the perpetrator who gets bullied (Wolke & Lereya, 2015). The term bully-victimization is synonymously used for both victims and the perpetrator who gets bullied (Alavi *et al.,* 2015; Fanti & Georgiou, 2013). In our study, bullying–victimization refers to any adolescent who had been bullied. Bullying among adolescents can occur in multiple forms as verbal bullying, physical bullying, sexual bullying and cyber-bullying. Verbal bullying includes name-calling, insults, teasing, intimidation, homophobic or racist remarks, or verbal abuse (Goodboy *et al*., 2016). Physical bullying includes hitting, pushing, kicking, choking and forcefully taking something from the victim (Kapoor *et al*., 2016). Sexual bullying is a type of harassment that occurs in connection with a person’s sex, body, sexual orientation, or sexual activity and can be physical, verbal or emotional (Fredland, 2008). Cyber-bullying is bullying with the use of digital technologies. It can occur on social media, messaging platforms, gaming platforms and mobile phones (Betts *et al*., 2017).

According to the Global Burden of Disease study 2018, bullying victimization is among the leading risk factors for mental disorders among adolescents (UNESCO, 2018). It is considered a contributing factor for depression amongst adolescents and children (Kelleher *et al*., 2008). A few studies have identified bullying victimization as one of the significant risk factors for anxiety disorders (Copeland *et al*., 2013; Silberg *et al*., 2016). A meta-analysis of cross-sectional studies reported that bullying victimization was significantly associated with depression, loneliness, reduced self-esteem, and self-concept (Hawker & Boulton, 2000). Bullying victimization is a risk factor for suicides, suicide ideation, and attempts among adolescents (Bang & Park, 2017; Barzilay *et al*., 2017). Regarding age, bullying has been reported to follow an inverted “u-shaped” curve with an initial peak at the age of 14 years and then a decrease. Males are more bullied than females (Silva *et al*., 2013). Bullying increases adolescents’ risk of falling into high-risk behaviours like smoking, drinking, and using drugs (González-Chica *et al*., 2019). Factors such as positive school climate, having friends at school, and good academic performances were reported as protective factors for bullying (Xu *et al*., 2020). Attributes such as low self-esteem and internalizing personality were positively associated with bullying-victimization (Slee & Rigby, 1993). Parental protective factors like positive upbringing, care, and protection were negatively associated with bullying (Shetgiri *et al*., 2013).

The socio-ecological diathesis stress model is designed to address bullying-victimization, the model explains the complex interaction of the socio-ecological model (socio-ecological inter connections in the child’s world) with the diathesis stress model which allows the understanding of the risk and protective factors of bullying. Hence this model helps to throw light upon bullying and its associated factors

Globally, the estimated prevalence of bullying victimization ranges from 10% to 44.6% among adolescents (Dew *et al*., 2008; Rigby & Slee, 1991). The highest prevalence was observed in the Eastern Mediterranean region (44.3–46.0%) and the African region (43.0–44.3%), and the lowest was observed in the European region, 8.0–9.0% (Biswas *et al*., 2020). In the Netherlands, nearly 17% were considered victimized (Jansen *et al*., 2012). In a study in the United States, 11% reported being the victims of bullying (Nansel *et al*., 2001).

India has the third-highest bullying rate among South-Asian countries (LLLFoundation, 2017). Studies in North India revealed a bullying victimization rate of 19.2% to 31.4%. The South Indian states of Tamil Nadu and Andhra Pradesh reported a prevalence of 16% and 13.4%, respectively, for bullying victimization (Narayanan & Betts, 2014; Nguyen *et al*., 2020)

Kerala, the most advanced Indian state in terms of epidemiological and demographic transition, reported one of the highest rates of depressive disorders (Dandona *et al*., 2017). It has a crude disability adjusted life years (DALY) rate of 646 mental disorders per 100,000 of the population, compared to an all-India average of 550 (Sagar *et al*., 2020). Despite its better performance in several health indicators, the state reports one of the highest sex and age-standardized suicide death rates of 28.3 per 100,000 males, compared to the Indian average of 21.2 (Sagar *et al*., 2020). Despite being a significant predictor of poor mental health, bullying is rarely studied in Kerala. Hence, we conducted a study to find the prevalence and factors associated with bullying victimization among adolescents in the Kozhikode district of Kerala.

## Methods

### Approach and research paradigm

We used a pragmatic paradigm to guide the mixed method study; the pragmatist uses different approaches to solve the problem and focuses on what and how of the problem (Creswell, 2014). The research design is a sequential explanatory design, where the quantitative phase is a cross sectional design and the qualitative phase is based on phenomenology.

### Researcher’s role

The researcher (Bhagiaswari Kodapally) conducted the study as a Master’s thesis for the Masters in Public Health program. She is a dentist and trained in both quantitative and qualitative research techniques. In the quantitative study the researcher played an insider’s role as she was the primary agent for data collection, analysis and interpretation. The researcher’s role as insider in the quantitative phase allowed her to gain knowledge on the real problem of bullying and this helped her frame the in depth interview guide for the qualitative phase. In the qualitative phase, the researcher played an outsider role, because the participants had no past association with the researcher and thus this role helped her to understand the participant’s perspective in a new dimension, which therefore eliminated any chance of interviewer bias.

### Context

The study targeted students of grades VII, VIII, and IX from four public schools in the Kozhikode district. The Kozhikode district is in the Kerala state of India. The state has a population of 3,089,543. The city is 38.25% urbanised and contributes up to 12% of the state’s income. The state literacy rate is 95.08%, with the school enrolment for the year 2018–19 at 389,497. The district has an educational index of 0.945 and human development index of 0.789.

### Sampling strategy

In the quantitative phase, we conducted a cross-sectional survey among students of grades VII, VIII, and IX from four public schools in the Kozhikode district. There were 56 public schools in the district, 30 funded and managed by the government (government schools) and 26 sponsored by the government and managed by a private partner (aided schools). The sample size was estimated using the formula N = (Z^2^ P (1-P)/ d^2^) * 2, where N = sample size, Z is the critical value for 95% confidence level, P is the anticipated prevalence (31.4%), and d is the half-width of the 95% confidence interval (precision) which was taken as 0.05. The sample size calculated was 662. After considering a non-response rate of 10%, we estimated a total sample size of 728. Considering that there are about 30 students in each class division, we expected to obtain a sufficient number of students from 24 divisions. The students were selected through a multistage cluster sampling approach.

In the first stage, we selected all the 56 schools funded by the government, i.e., 30 government schools and 26 aided schools. In the second stage, we randomly selected two schools, two each from the government schools (two out of 30) and the aided schools (two out of 26). In the third stage, we randomly selected two class divisions of grades VII, VIII, and IX from each of the four selected schools, making a total of 24 class divisions out of 78 divisions. Each class had 30 to 40 students. All the male and female students of grades VII, VIII, and IX in the above schools were eligible for the study. Students from private schools and those who were not physically present during the survey were excluded. A total of 854 students were approached for the study. The data were collected from a sample of 764 students after obtaining necessary approvals from students and parents.

In the qualitative phase, purposeful sampling was performed from the database of the cross-sectional survey participants to identify the key informants for the qualitative study. In-depth interviews were conducted among four key informants, namely, one class teacher (female), two male adolescents aged 13 years (bully-victim), and a female parent (mother of a bully-victim). Verbal consent was obtained to take part in the in-depth interview. The in-depth interview guide is in Table 1.

### Permissions and ethical clearance

Prior permission was obtained from the deputy director of the education department to survey schools in the Kozhikode district. The ethics approval was obtained from the Institutional Human Ethics Committee (IHEC) of Central University of Kerala, Kasaragod (Reference No: CUK/IHEC/2019/036). An informed consent form and the participant information sheet were given to students to obtain signed consent from parents at home. Those students who brought back signed informed consent from the parents were given informed assent forms to sign before their inclusion in the study. In total, 90 parents (i.e., 9.01 %) did not grant permission to participate in the research, and those students were excluded from the study. The identities of students and schools were masked to ensure privacy and confidentiality. For the in-depth interview, verbal informed consent was obtained from the school administrator, the class teacher, the parent, and the parents of two students. Verbal assent was obtained from the two students.

## Data collection procedure

### Quantitative (cross-sectional study)

Bullying victimization was captured using a 60 item questionnaire that consisted of questions from the revised Olweus Bully-Victim Questionnaire (OBVQ), the most frequently used self-report measure of bullying and victimization experiences (Solberg & Olweus, 2003). The item “How often have you been bullied at school in the past couple of months?” is used to capture bullying victimization. The responses for the question were: 1) Haven’t been bullied in the school in the past couple of months; 2) It has only happened once in a month; and 3) It has happened more than once in a month. It has been asserted that being bullied more than once in a month was a suitable lower-boundary cut-off for differentiating the children who are frequently bullied from those who are not (Solberg & Olweus, 2003). This tool was validated in India (Verman, 2017). The socio-demographic variables, psycho-social factors, health behaviours, risk behaviours, and protective factors were captured using the modified country-specific Global School Health Survey questionnaire (World Health Organisation, 2013). Depression was captured using Patient Health Questionnaire (PHQ-9), validated in India. A pilot survey was performed among ten school students who were not included in this study, between the ages of 12 to 14 years, to understand the comprehensibility and feasibility of the data collection tool. Minor modifications were made to the questionnaire after the feedback from the students. An open-ended question on the participant’s mood was modified by adding a list of responses, converting it to a closed-ended question.

Before data collection, the researcher explained the study’s need and their role in the study. The participant information sheet and the informed consent form were given to students before obtaining signed consent from parents at home. The eligible consented students participated in the survey. Approximately 60 minutes were given to complete the questionnaire. The participants were asked to recall their life and school experiences in the past couple of months (i.e., the beginning of the academic year June to December 2019, to the day of data collection).

### Qualitative study

Participants were briefed on the purpose of the in-depth interview, and informed consent was taken for recording and participation. The interview was done using an in-depth interview guide (Table 1), which lasted for 45–60 minutes and was audio-recorded. The questions were asked in the vernacular language (*Malayalam*) and transcribed. The transcripts were translated to English and back-translated for quality and accuracy. The transcripts were reviewed and approved by a language expert. To ensure the trustworthiness and credibility of the qualitative data analysis, peer debriefing was done on a continuous basis with a qualitative research expert, who is not part of the study. A conventional Braun & Clark’s thematic analysis was adopted to analyse the data in Nvivo version 1.5. An open-source alternative for Nvivo could be RQDA, an r package for qualitative analysis. The inductive coding was done in preliminary stages, and in further revisions, deductive codes were applied. Similar codes were identified and classified; similar categories were identified to form the themes reviewed, named, and defined. The results were categorized into major themes.

### Statistical analysis

The statistical analysis for quantitative data was carried out in the SPSS software (IBM Corp), version 25.0. Data cleaning was performed in order to address the missing values, outliers, and inconsistent values in the data. The outcome variable for the study was bullying (i.e., children who reported bullying more than once a month). We classified the responses to the question “How often have you been bullied at school in the past couple of months?” into two categories to get a nominal category. We coded the options “Didn’t get bullied in the past couple of months” + “Bullying happened only once in a month” as ‘0 = No’ and “Bullying occurred more than once in a month” as ‘1= Yes’, which was the established cut off for the participant to be a victim (Smith *et al*., 2008). In univariate analysis, the variables were presented with frequency (prevalence), means, and standard deviations. In bivariate analysis, victim-associated factors and bully-associated factors were identified. Variables which were significant with a ‘p’ value of less than 0.05 were included in the binary logistic regression analysis. The factors identified through binary logistic regression were reported as two groups: 1) The factors related to bullying victimization; and 2) The perception of the victim of the bully and the school.

## Results

### Quantitative analysis

Out of 854 students approached, 764 students gave consent and completed the self-administered questionnaire (response rate 93.1%). Table 2 describes the socio-demographic characteristics of the study sample.

**Figure 1:**
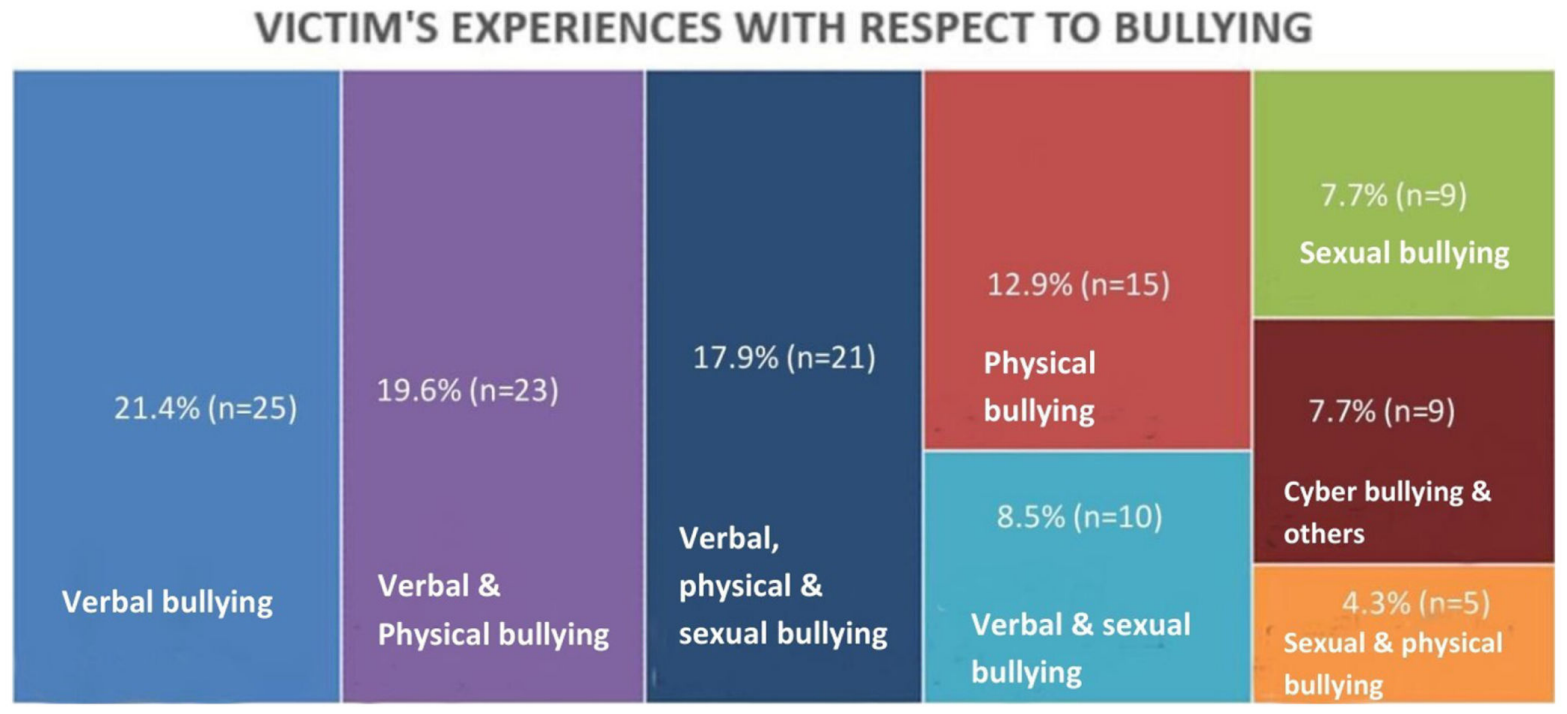
Categorization of the types of bullying combinations experienced by the victims of bullying. *Others – include the combinations (verbal and cyber bullying, physical and cyber bullying, sexual and cyber bullying, verbal + physical+ sexual and cyber bullying, verbal+ physical and cyber bullying, verbal + sexual and cyber bullying, physical+ sexual and cyber bullying, and only cyber bullying).

Bullying was captured using the revised OBVQ; an adolescent is considered a victim of bullying if they reported experiencing bullying more than once in a month in the past couple of months (Smith *et al*., 2008). About 15.3% (n=117) of the students reported being bullied more than once in a month. The majority of them were boys (n=80). At least two in every 10 boys and one in every 10 girls in high school get bullied at school. Moreover, among adolescents who reported being bullied more than once in a month, multiple experiences of a single form of bullying and multiple forms of bullying were observed. The categorization of the types of bullying combinations experienced by victims of bullying is given in Figure 1.

Among the adolescents who were identified to have experienced bullying more than once a month (n=117), it was found that more than 60 % (n=74) experienced more than one form of bullying. In comparison, both verbal and physical bullying were experienced by 19.6 % (n=23) of the adolescents. The trio of verbal, physical, and sexual bullying was experienced by 17.9% (n=21). It was also often observed that the growing phenomenon of cyber-bullying co-existed with other forms of bullying 7.7 % (n=9), though it did not exist alone (n=0).

The chi-square test was done to find the variables significantly associated with bullying at P-value < 0.05. Two crucial categories were identified: 1) The factors related to bullying victimization, including the demographic, socioeconomic, psychological, risk behaviours and home environment of the participants; and 2) factors related to the victim’s perception of the bully and the school environment. The significantly associated factors in bivariate analysis were included as the independent variable for multivariate analysis (Binary logistic regression). Table 3 and Table 4 describe the factors associated with bullying victimization.

The factors like age and sex, the participant’s mood, feeling worthless, having depression, participant happiness at home, receiving harsh punishment at home, the parents’ and victims’ response to bullying were all significantly associated with bullying. Bully and school-related factors as perceived by the victim were the sex of the bully, place of bullying, perceived reason behind the intention of bullying, the teachers’ response to bullying, and unsafe school environment; all were significantly associated with bullying.

Binary logistic regression was used to quantify the effect of independent variables on bullying. The variables found to be significantly associated with bullying at P-value < 0.05 in bivariate analysis (chi-square tests) were included as independent variables in the model. Binary logistic regression yielded a significant model with an acceptable model fit. Adjusted odds of being bullied with 95% confidence intervals (CI) were computed. Adjusted and unadjusted odds ratios (OR) were reported for bullying victimization factors and the bully and school-related factors Factors such as depression, school action against the bully, and unsafe school environment were not found to be significantly associated (P>0.05). The factors associated considerably with bullying are presented in Table 5 and Table 6.

Individuals of the male sex had an increased likelihood of being bullied and being a bully. Similarly, the likelihood of being bullied was higher among children who reported being sad, moody, and have a feeling of worthlessness. The parental response and the victim’s response to bullying increased the likelihood of bullying. Similarly, the teacher taking action against the bully’s bully mentality and being in the playground was found to increase bullying.

### Qualitative analysis

The key findings from the qualitative research were categorized into major themes and subthemes.

#### Theme 1: family environment

##### Parental interaction with the children

The children of overprotective parents were more easily targeted by the bullies and had less competent social skills. Though they may have a positively adjusted familial relationship, they were most likely to suffer from social anxiety issues, thus making them easy prey for bullies.

“There is nothing my child hides from me. I make it a point to talk to the school authorities when needed.” – Mother of a bully victim

Similarly, advocates of permissive parenting or blind parenting support their children in all circumstances, thereby raising a bully. Parents of victims and class teachers reported reluctance on the part of parents of bullies.

“**I** know the mother of a bully, who worked in his interest and used to hide these school issues from his dad.” - Mother of a bully victim

“We call their parents first, but the irony is parents never tend to accept their child as a bully.” -Class teacher

#### Theme 2: School environment

##### Role of class teacher/school authorities

The readiness of the teacher to intervene plays a significant role in curbing bullying practice. The teacher’s response to bullying may vary depending on the beliefs and attitude of the teacher. Reportedly there were two types of teachers, the ones who intervene, either through corporal punishment or counselling the bully or reporting to parents and the others who never bother to intervene.

Bullying is seen as culturally normative behaviour, especially among boys. Lack of awareness on bullying as unhealthy behaviour, limited competence to detect a bully, the heavy academic workload of teachers, and the assumption that they will outgrow bullying were the commonly cited reasons for not intervening in the bully behaviour.

“Some of the teachers find it difficult to understand these difficult children and leads to criticizing the child and blaming them for their aggressive behaviours, they feel such children are untameable, and no amount of effort would change them.” - Female class teacher

##### Corporal punishment

Corporal punishment and expulsion from school were ineffective in curbing bullying as there seems no modification on the social behaviour. Moreover, it was observed that schools feared that any corporal punishment, aside from being ineffective, would attract negative publicity to the school.

“Another issue is the involvement of the child rights commission, who blame the school for taking action against the bully, and these issues would attract media creating havoc and negative publicity. What happens is that, after making so much of issues, the school has to take the bully back “- Class teacher

##### The anti-bullying policies

The schools were found to have no specific anti-bullying policies or monitoring mechanisms. However, it was observed that some of the teachers had training sessions on child psychology under a Non-Government Organisation (NGO) called Urban Resource Centres. Parents were aware of the supportive programs at the school. Moreover, the parents were also aware of such training and mentorship programs.

“This project is called ORC (Our Responsibility to Children). We pay a good amount for these classes where the psychologist is paid in hours. The money is collected from these students for the classes.” - Mother of a bully victim

“As a part of URC, we have a ten-day training program on mentorship during the summer vacations. Here the teachers are taught how to deal with children. This training program is compulsory.” - Class teacher

#### Theme 3: The victim perspective

The victims mostly perceived that bullying can be tackled by them and found it silly to report to their parents or teachers. Moreover, an underlying perception of reporting bullying to parents and teachers as a sign of weakness was observed, as victims complained of being teased by their peers.

“Mom always takes these things seriously. Hence, she reports it to the teacher, and then she follows up if I have any further issues. But most of the time I try to tackle it by myself; otherwise my classmates would tease me.” - Bully victim 1

“They are just my friends teasing me, even though I feel a little bad, but I know they are joking. I tackle these issues by myself.” – Bully victim 2

These incidents do traumatize them, and later the children get adjusted to it. At some point, the children accept the bullying, and a sense of helplessness develops in them as they think the bully is more powerful than them.

“They are big influential group and children like us don’t have any support” - Bully victim 1

**Figure 2:**
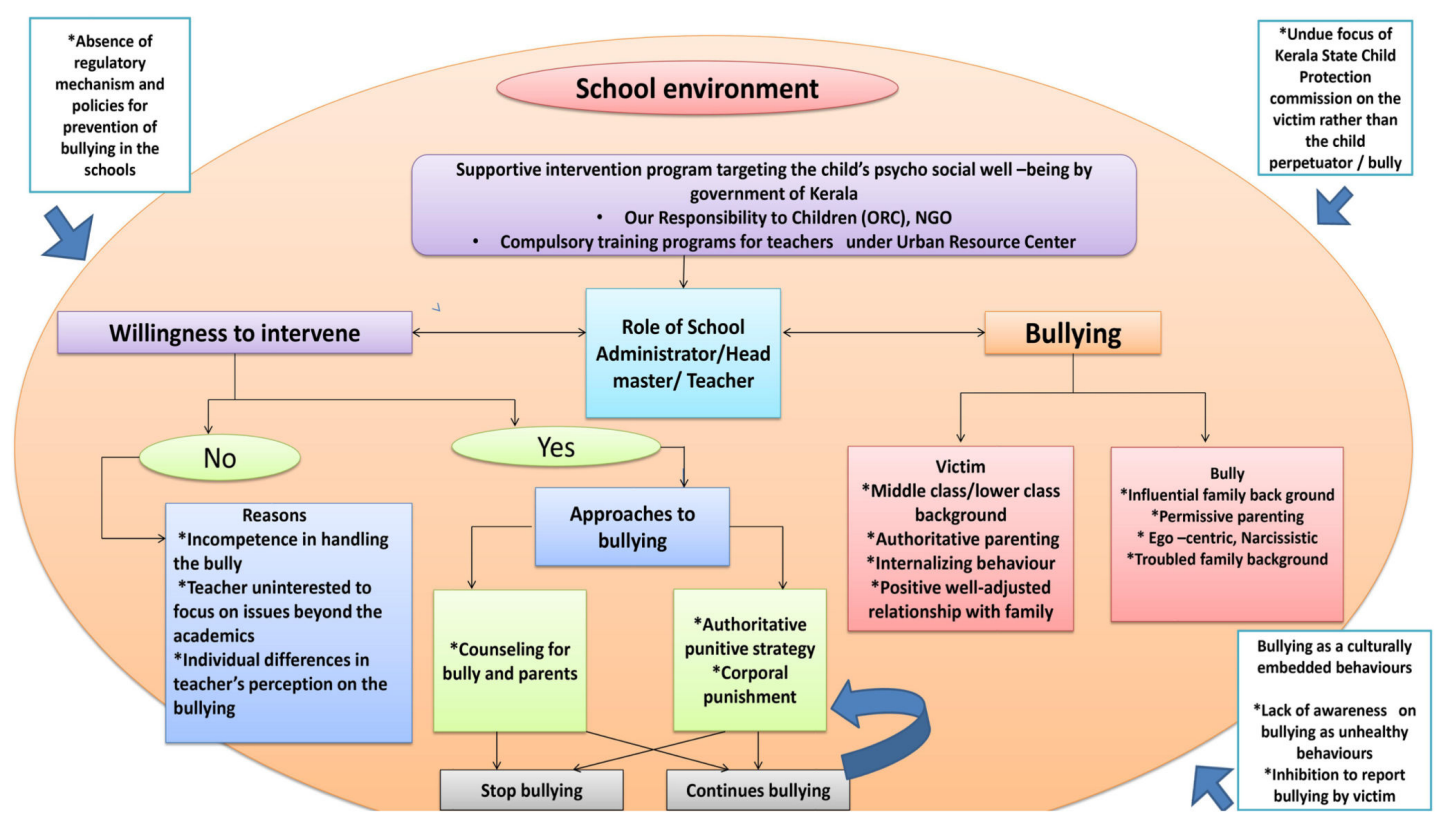
Pathways of bully-victimization among adolescents.

The pathways of bully victimization among adolescents, are given in Figure 2

## Discussion

This study aimed to measure the prevalence of bullying-victimization in public schools of the Kozhikode district, Kerala, and to find out the factors associated with bullying. The prevalence of bullying victimization among high school students from grades seven to nine was estimated as 15.3%, similar to a study from the neighbouring state of Tamil Nadu (Narayanan & Betts, 2014). The prevalence of bullying victimization in studies from North India ranged from 16% to 24 % (Rana *et al*., 2020; Thakkar *et al*., 2020b). The lower prevalence in our study could be attributed to the heterogeneity of the study settings in India and under-reporting due to lack of awareness of bullying (Charak & Koot, 2015). The current study considered a child to be the victim of a bully if they reported experiencing bullying more than once a month in the past couple of months. The consideration of time and persistence in operationalizing bullying could be one of the reasons for the lesser prevalence of bullying reported in our study (Chen & Cheng, 2013). The evidence says that only a small percentage of children, i.e., 1.6% to 15%, are persistently bullied, reflecting that our findings align with other previous studies conducted (Craig & Pepler, 2003; Solberg & Olweus, 2003).

Increasing age was significantly associated with bullying in this study; bullying intends to peak at 14 years (56.5%) of age, similar to another study in India (Rana *et al*., 2020) and studies undertaken in the USA and Canada (USDHHS, 2017; Vaillancourt *et al*., 2010). Adolescence is a sensitive life period characterized by social, emotional, and psychological changes. Bullying is more targeted and persistent during this phase, and the strong involvement of cultural context plays a significant role in increasing age is a significant factor of bullying (Malta *et al.,* 2014)

Our study found more boys are getting engaged in bullying victimization (76%) and are perpetrators of bullying (81.2%) than the girls, which is akin to the findings from other studies both nationally and internationally. A cross-country study across 40 countries reported bullying as higher among boys (ranging from 8.6% to 45.2%) than girls (4.8% to 35.8 %) (Craig *et al*., 2009). Another study from India revealed that boys were more likely to be bullied and 2.25 times more likely to be perpetrators than girls (Malhi *et al*., 2014). The qualitative findings showed that boys try to tackle the problem by themselves and find it petty to inform elders about being bullied. This can be attributed to the culture of boys being projected as masculine, and seeking help from others is seen as a sign of weakness in front of peers (Ranjith *et al*., 2019)

Among those who were bullied, verbal bullying was common among both genders. Boys were more into physical bullying (69.7%) while girls were more into verbal bullying (82.1%); these findings were similar to an earlier study from South India (Kshirsagar *et al.,* 2007). Prevalence of sexual bullying was seen more in males (42.7%) in our study. However, most studies showed the prevalence of sexual bullying more in females (Kepenekci & Cinkir, 2006; Pengpid & Peltzer, 2013). This could be explained by a report from Times of India that stated Kerala has the highest number of child abuse cases in India, out of which 85% were boys. Another rising trend observed is cyber-bullying (7.7%), which could be a concern in the growing digital era. Though it didn’t stand alone, it was observed that cyber-bullying co-existed with other forms of bullying. Increased digitalization, accessibility of smartphones, and affordable internet could potentially increase cyber-bullying in the future. Moreover, children who might have been bullied verbally, physically, or through other forms might also be at high risk of cyber-bullying. Additionally, our study also reveals how the same victim suffers various types of bullying, as presented by other studies (Craig & Pepler, 2003)

When psycho-social characteristics were assessed, feeling sad and moody increased the significance of being bullied (23.9%). Our finding is in line with another study that reported that withdrawn and irritable children are more prone to be victims, as they are more likely to have internalizing behaviours like fear of failure, anxiety, and feeling of worthlessness (24.8%), which was also found to be significantly associated with bullying victimization in the current study (Gendron *et al*., 2011). Qualitative findings in this study suggested that the bullies more easily targeted the children of overprotective parents. This could be because of poor social skills, leading to poor social adjustments and anxiety issues, making them an easy target for the perpetrator (Efobi & Nwokolo, 2014; Kokkinos, 2013). Similarly, permissive parenting increases the likelihood of the child being a bully, as these children get undeniable support from parents even when they project anti-social behaviours. Other studies, too, have reported the permissive parenting style as a critical factor in adolescents becoming a bully (Efobi & Nwokolo, 2014).

Harsh punishments at home increased the likelihood of bully victimization, similar to another study (Jaghoory & Björkqvist, 2013). Children who are disciplined in an authoritarian manner tend to receive severe penalties at home, leading to them internalizing behaviours. These children become moody, insecure, and less confident, making them easy prey for the bully (Garcia & Gracia, 2009).

Whether the victim’s parents are taking any actions against the act of bullying was found to likely increase the odds of being bullied. Though this is very unlikely to happen, studies support how children experience bullying despite reporting it to elders (Brown *et al*., 2013). Bullying is mostly not recognized as an unhealthy behaviour and, to some extent, is a culturally acceptable normative behaviour. In the qualitative exploration, we found that parent’s response to the bullying victimization of their child did not prevent them from being bullied further. These events make the victims helpless, traumatized, submissive, and they get accustomed to bullying. This self-acceptance of the child as a victim would significantly hamper their mental, emotional and social well-being (Uba *et al*., 2010)

The most common place for bullying is the playground. It is where the students interact the most, away from any kind of supervision, thereby creating a conducive environment for bullying. The thought of seeking happiness through seeing others hurt was the perceived reason behind most of the bullying incidents in the views of the victims; these findings are in line with another study in India that reveals a non-pathologic narcissistic attitude predicts a bully (Thakkar *et al*., 2020a).

At the school level, the teacher trying to intervene with the bully was seen to increase the odds of bullying. The initial response to bullying from school authorities was corporal punishment. However, corporal punishments are known to be transient and often ineffective in curbing bullying. Supportive involvement of the bully’s parents is a crucial factor in reducing bullying behaviour. In our study, it is reported that most of the parents have a cold attitude toward the school as they don’t accept their children as bullies. In turn, they blame the school authorities for punishing their child, creating havoc by involving the Child Rights Commission (a social welfare initiative under the Government of Kerala to protect children). This is when the bully has the advantage of having undivided support from the parents and the regulatory agency such as the child rights commission, which unknowingly creates a protective guard for the bullies. Due to the fear of spoiling the school’s reputation, the school management gives ineffective, transitory punishments. This leads to the bully becoming the ultimate ‘winner’, who realizes they cannot be touched, thereby attaining the power and impeccable sense of security, making them stronger to the extent they start bullying the teachers (Biswas *et al*., 2020). Organizations like the Child Rights Commission are more victims oriented. They do not address a child perpetrator (for example, considering the school authority as a perpetrator, as per the complaint registered by the bully’s parents against the school).

The unwillingness of teachers to intervene with a bully is another concern. Factors include a lack of empathy for both the bully and the victim, inability to identify the bully or victim, the teacher considering bullying as normative behaviour, a mishandling or lack of competence in dealing with a bully, a heavy academic workload and a fear of the bully harming them. (De Luca *et al*., 2019)

At the macro level, society fails to identify bullying as an unhealthy behaviour as it is deeply embedded in the culture, to the extent that bullying is considered a part of growing up. It’s high time we change the idea that “the children are like this only; it’s only because of their age”.

At the policy level, there are few child-centric training programs for teachers under Urban Resource Centres, an NGO in collaboration with the Government of Kerala. However, these training sessions do not address the management of bullying practices; instead, they concentrate on helping teachers manage an academically weak student and managing a difficult child. Similarly, Our Responsibility to Children (ORC) is another NGO organization supported by the Government of Kerala that generally focuses on the social welfare of the students. The parents also attend these sessions to learn the ways of parenting. These are the support mechanism currently available that generally addresses improving the psycho-social well-being of the children as a whole and does not mainly target the outliers in terms of behaviours. Hence, to target the particular outliers, you need a specialized training session for all key stakeholders (parents, teachers, and students).

We learned that India has no policies or laws to address bullying behaviour at the school level, unlike the anti-ragging policies at the graduate level. In 2015, the Central Board of Secondary Education (CBSE) issued guidelines for bullying prevention, including the mandatory setting up of anti-bullying committees in schools (Rai, 2019). The committee aimed to develop and implement bullying prevention programs, training programs for staff, students, and parents, and create awareness through various programs. In the state of Kerala, the Raghavan committee report recommended that teachers and the principal be held liable if any act of bullying takes place in the school premises (Pandey, 2017). With no proper rules and regulations, schools in Kerala practice the same old traditional method of corporal punishment to curb bullying with little to no effect.

One of the limitations of the study is the self-reporting of bullying, which may lead to reporting bias. However, the bias is limited to a great extent by using a validated self-administered questionnaire.

Bullying needs multi-disciplinary effort, right from the government at the apex to the children at the base. A major recommendation from this study is that anti-bullying policies need to be laid at the apex level and implemented at all schools. A comprehensive, supportive, cooperative, and sustainable bullying prevention program needs to be included in the curriculum, targeting the young population at school. Training should be given to non-teaching staff at the grassroots level to identify bullying and report it to the school authorities. Anti-bullying helplines in schools can be initiated to facilitate bullying prevention. Moreover, teachers should be trained to identify bullying victims and tackle bullying with sensitivity.

The schoolchildren’s mental health and well-being are critical, particularly in a state like Kerala, which has one of the highest DALY rates of mental disorders in India. As the recent science editorial (Leshner, 2021) wrote, this needs to be addressed very seriously by the educational authorities. The mere provision of a few psychologists or some psychology sessions may not be sufficient to manage them. The positive mental health of children is essential not only for the future health of the children but also for society as a whole.

## Supplementary Material

Supplementary Material

## Figures and Tables

**Table 1:** In-depth interview guide.

Category of respondent	Questions	Probe questions
School authority/class teacher	Have you heard of the term of bullying?	
	Did you have any experiences in the class with bullying behaviour among adolescents?	a) How did you manage it? b) How did the bully respond to you when you confronted him? c) How did you pacify the victim? d) How do you think teachers tackle bullying?
	How does the school support in managing a bully?	a) What action did the Headmaster take against the bully? b) What are the restrictions you are talking about?
	What do you think is the stance of the parents?	a) Why do you think the parents were hesitant to take their child for counselling?
	Do you have anti bullying policies at school?	a) Please explain in detail what all training sessions did you get in managing a difficult child as the part of teachers training session?
The bully-victim	Have you heard of the term bullying?	
	Do you have any experience of being bullied?	a) How did you respond when bullied?
	What is the normal course of action when bullying is reported?	a) Please explain how the school authorities reacted to the reporting, especially your class teacher and School head? b) Did the teachers pacify you? c) Are your teachers and school head supportive?
	Are there any bullies in your class who continues to bully even after prompt action is taken?	a) Why do you think they still continue bullying? b) Aren't they afraid of any punishments?
	Did you report bullying to your parents?	a) How did your parents react to you being bullied?
	How is your relationship with your parents?	a) Do you want to share anything else?
The mother of the bully victim	Have you heard of the term bullying?	
	Did your child ever report being bullied to you?	a) Please share the incidents in detail
	What did you do about it?	a) What was the response of the school authority to bullying b) Do the parents of children who have experienced similar episodes of bullying talk about this?
	What is the attitude of the parents of bully?	a) How badly do you think the parental influence have affected the bully?
	Do you know any anti-bullying policies followed in school?	a) As a parent how effective do you believe the Our Responsibility to Children (ORC) program is for you?
	How is your relationship with your child?	a) Do you have anything else to say?

**Table 2:** Socio-demographic characteristics of the sample (N=764).

Variables	Categories	Frequency	Percentage (%)
Age in years	Less than 14 years	433	56.7
	14 years and above	331	43.3
Sex of the participant	Male	447	58.5
	Female	317	41.5
Siblings	No siblings	33	4.3
	One sibling	266	34.8
	More than one sibling	465	60.9
Father’s education	< 10 Years of schooling	178	22.6
	>=10 years of schooling	586	77.4
Mother’s Education	< 10 years of schooling	57	7.5
	>=10 years of schooling	707	92.5
Father’s occupation	Unskilled	290	38
	Skilled	474	62
Mother’s occupation	Unskilled	692	90.6
	Skilled	72	9.4
Ration card type	Above poverty line	267	34.9
	Below poverty Line	497	65.1
Grade enrolled			
	7^th^ standard	209	27.4
	8th standard	272	35.6
	9th standard	283	37.0

**Table 3:** Factors associated with bullying victimization: results of bivariate analysis (n=764).

Independent variable	Category	Being bullied Yes n (%)	Unadjusted OR (95%CI)	P value
Age	Less than 14 years	51(11.8)	Ref	
	14 years or more	66(19.9)	1.87(1.25-2.78)	<0.001*
Sex	Female	28(8.8)	Ref	
	Male	89 (19.9)	2.56(1.63-4.03)	<0.001*
Mother's education	Up to 10^th^ standard	12(14.6)	Ref	
	10^th^ standard or higher	105(15.4)	1.06(0.56-2.02)	0.850
Having best friends (can be in school, at home etc.)	Yes	108(15)	Ref	
	No	9(21.4)	1.56(0.72-3.33)	0.258
Mood of the Participant most of the time	Those who are mostly happy	89(13.5)	Ref	
	Those who are mostly moody or sad	28(26.7)	2.33(1.43-3.79)	0.04*
Depression	No	94(14)	Ref	
	Yes	23(25)	2.05(1.21-3.44)	0.006*
Ever felt worthless during the past two weeks	Not at all	88(12.8)	Ref	
	Most of the days	29 (39.2)	4.41(2.63-7.4)	<0.001*
Tobacco smoking	No	108(14.8%)	Ref	
	Yes	9(25%)	1.91(0.88-4.18)	0.150
Harsh punishment from home	Didn't receive	92 (13.4)	Ref	
	Received	25 (32.9)	3.17 (1.88-5.38)	<0.001*
Parental response to their children being bullied	Didn't take any actions against the bullying	91(12.7)	Ref	
	Took action against the bullying	26 (55.3)	8.52 (4.60-15.76)	<0.001*
Victims response to bullying	Did not react to bullying	73 (10.7)	Ref	
	Reacted to bullying	44 (55.7)	10.5 (6.35- 17.4)	<0.001*

**Table 4:** Factors associated with bullying victimization (the perception of the victim on the bully and the school): results of bivariate analysis (n=764).

Independent variable	Category	Being bullied Yes n (%)	Unadjusted OR (95%CI)	P value
Sex of bully	Females	22(13)	Ref	
	Males	95(16)	1.27(0.77- 2.09)	<0.348
Place of bullying	School premises	82(11.5)	Ref	
	Playground	35(71.4)	14.04 (9.96-37.38)	<0.001*
Perceived reason behind the intention of bullying	For joke	49(7.7)	Ref	
	Seeking happiness in hurting others	68(54.4)	14.36(9.09-22.04)	<0.001*
School action against the bullying	Didn’t take any action	99(14.3)	Ref	
	Took action	18(25.7)	0.48(0.27-0.86)	0.011*
Teachers reaction to bullying	Didn’t take any action	79(12)	Ref	
	Took some action against the bullying	38(36.2)	4.16 (2.62-6.61)	<0.001*
Academic performances	Below average	7(14.9)	Ref	
	Average and above	110(15.3)	1.03(0.45-2.37)	0.934
Unsafe school environment	No	107 (14.6)	Ref	
	Yes	10 (33.3)	2.93 (1.33-6.43)	0.01*

**Table 5:** Factors associated with bullying victimization: results of binary logistic regression analysis.

Independent variable	Sub categories	Adjusted OR (95%CI)	P value
Age	Less than 14 years	Ref	
	14 years or more	2.09 (1.31–3.32)	0.02*
Sex	Female	Ref	
	Male	3.50 (1.97–5.87)	<0.001*
Mood of the participant, most of the time	Those who are mostly happy	Ref	
	Those who are mostly moody and sad	2.06 (1. 12-3.77)	0.019*
Ever felt worthless during the past two weeks	Not at all	Ref	
	Most of the days	2.83 (1.30–6.14)	0.008*
Victims response to bullying	Did not react to bullying	Ref	
	Reacted to bullying	8.01(4.53–14.36)	<0.001*
Harsh punishment from home	Didn’t receive	Ref	
	Received	2.18 (1.14–4.17)	0.017*
Parental response to their children being bullied	Didn’t take any action	Ref	
	Took action	5.27 (2.44–11.36)	<0.001*

* **Dependent variable**: Child was bullied at least twice in the month (yes or no), *No = Reference Category* ^*^Variables in the model: age of participant, sex of the participants, parent response to bully, victim reaction to bully, mood of the participant most of the time, punishment from home, feeling worthless, and depression

**Table 6:** Factors associated with perception of the victim of the bully and the school: results of binary logistic regression analysis.

Independent variable	Sub categories	Adjusted OR (95%CI)	P value
Sex of bully	Female	Ref	
	Male	2.19 (1.16-4.15)	0.015*
Perceived reason behind the intention of bullying	For joke	Ref	
	Seeking happiness in hurting others	10.23(6.14- 17.05)	<0.001*
Place of bullying	School premises	Ref	
	Playground	9.41(4.29-20.64)	<0.001*
Teachers reaction to bully	Didn’t take any action	Ref	
	Took some action against the bully	3.59 (1.91-6.73)	<0.001*

* **Dependent variable:** Child was bullied at least twice in the month (yes or no), *No = Reference Category*

∞ Variables in the model: place of bullying, teachers reaction to bullying, school action against bullying, sex of bully, and perceived reason behind the intention of bullying.

## Data Availability

OSF: Bullying victimization among adolescents. https://doi.org/10.17605/OSF.IO/K42DY. This project contains the following underlying data: - Bullying victimization among Adolescents.csv (The data-set includes socio-demographic information and factors that are associated with bullying among adolescents aged 12–14years) - Transcripts of the qualitative component OSF: Bullying victimization among adolescents. https://doi.org/10.17605/OSF.IO/K42DY. This project contains the following extended data: - Participant information sheet, Informed consent, assent form.pdf - Study tool.pdf - Data set and Codebook.csv (Codebook for the data set) Data are available under the terms of the Creative Commons Attribution 4.0 International license (CC-BY 4.0).
