# Supplementary material for: Bullying victimization and its associated factors among adolescents in Kozhikode district, Kerala, India: a mixed-methods study [version 1; peer review: 1 approved]
Source: Wellcome Open Res. Author manuscript; Available in PMC 2022 Jun 10. (PMC7612838; doi:10.12688/wellcomeopenres.17102.1)
Supplement: Supplementary Material [file EMS145406-supplement-Supplementary_Material.pdf]

# Open Peer Review

## Current Peer Review Status:

Version 1

Reviewer Report 24 November 2021

https://doi.org/10.21956/wellcomeopenres.18889.r46612

**© 2021 Mehta M.** This is an open access peer review report distributed under the terms of the Creative Commons Attribution License, which permits unrestricted use, distribution, and reproduction in any medium, provided the original work is properly cited.

**Manju Mehta**
Department of Psychiatry, All India Institute of Medical Sciences, New Delhi, India

This article reflects bullying that adolescents have experienced. It is common in schools. It may have some serious consequences especially mental health like depression, poor self esteem and anxiety. They avoid going to school and afraid of other children. It may lead to poor academics also. The paper refers to types of bullying - verbal, physical, sexual and cyber. The most common is via social media, cyber. If the coping skills are not good then adolescent can be influenced badly.

- The article is well researched and written in detail.
- The introduction is long and covers all the points. Parent concerns are also addressed. The parents are more disturbed and worried. The authors have given themes.
- They have worked on govt. school children, but the responses of public school children could be different.
- The methodology is suitable for the population studied and the sample size is large enough. The tools used in data collection are valid. The analysis is also satisfactory.
- The references are satisfactory.
- The results and discussion is well written.
- The paper does not give details of role of teachers and increasing the coping skills of the adolescents.

I approve this article.

**Is the work clearly and accurately presented and does it cite the current literature?**
Yes

**Is the study design appropriate and is the work technically sound?**

Yes

**Are sufficient details of methods and analysis provided to allow replication by others?**
Yes

**If applicable, is the statistical analysis and its interpretation appropriate?**
Yes

**Are all the source data underlying the results available to ensure full reproducibility?**
Yes

**Are the conclusions drawn adequately supported by the results?**
Yes

***Competing Interests:*** No competing interests were disclosed.

***Reviewer Expertise:*** Clinical psychology child and adolescent mental health

**I confirm that I have read this submission and believe that I have an appropriate level of expertise to confirm that it is of an acceptable scientific standard.**
